# Leveraging clinical intuition to improve accuracy of phenotype-driven prioritization

**DOI:** 10.1016/j.gim.2024.101292

**Published:** 2024-10-10

**Authors:** Martha A. Beckwith, Daniel Danis, Yasemin Bridges, Julius O.B. Jacobsen, Damian Smedley, Peter N. Robinson

**Affiliations:** 1 The Jackson Laboratory for Genomic Medicine, Farmington, CT; 2 William Harvey Research Institute, Queen Mary University of London, London, United Kingdom; 3 Berlin Institute of Health, Charité-Universitätsmedizin Berlin, Berlin, Germany

**Keywords:** Genomic diagnostics, Human Phenotype Ontology, Likelihood ratio, Mendelian disease

## Abstract

**Purpose:**

Clinical intuition is commonly incorporated into the differential diagnosis as an assessment of the likelihood of candidate diagnoses based either on the patient population being seen in a specific clinic or on the signs and symptoms of the initial presentation. Algorithms to support diagnostic sequencing in individuals with a suspected rare genetic disease do not yet incorporate intuition and instead assume that each Mendelian disease has an equal pretest probability.

**Methods:**

The LIkelihood Ratio Interpretation of Clinical AbnormaLities (LIRICAL) algorithm calculates the likelihood ratio of clinical manifestations represented by Human Phenotype Ontology terms to rank candidate diagnoses. The initial version of LIRICAL assumed an equal pretest probability for each disease in its calculation of the posttest probability (where the test is diagnostic exome or genome sequencing). We introduce Clinical Intuition for Likelihood Ratios (ClintLR), an extension of the LIRICAL algorithm that boosts the pretest probability of groups of related diseases deemed to be more likely.

**Results:**

The average rank of the correct diagnosis in simulations using ClintLR showed a statistically significant improvement over a range of adjustment factors.

**Conclusion:**

ClintLR successfully encodes clinical intuition to improve ranking of rare diseases in diagnostic sequencing. ClintLR is freely available at https://github.com/TheJacksonLaboratory/ClintLR.

## Introduction

Next-generation sequencing (NGS) technologies, including exome sequencing and genome sequencing (GS), have had dramatic effects on the cost, accuracy, and utility of genetic testing.^1^ The overall diagnostic yield depends on the clinical cohort, but typically rates of 25% to 50% are reported.^2–4^ A major focus of translational genomics research is to improve the performance of NGS-based diagnostics in rare disease. Efforts are focusing on improving technologies used to ascertain sequence variants, including long-read sequencing, RNA-sequencing-based analysis, and optical genome mapping.^5–7^ Other efforts have focused on improving the performance of phenotype-driven prioritization, which leverages a comparison of the phenotypic abnormalities of the individual being investigated with those previously associated with human diseases or genetically modified model organisms to rank candidate variants.^8–10^ A number of methods have been published that apply statistical and machine learning algorithms to improve the performance of matching phenotypic features observed in a patient with features of Mendelian diseases.^11–17^

Computational methods for improving the yield of diagnostic NGS include phenotype-driven prioritization approaches that use the Human Phenotype Ontology (HPO) to record and analyze phenotypic abnormalities. The HPO is a comprehensive bioinformatic resource for the analysis of human diseases and phenotypes, offering a computational bridge between genome biology and clinical medicine, and is widely used for analysis and exchange of phenotype data in rare disease.^18–25^

To our knowledge, no available algorithm explicitly models clinical intuition. However, the clinical assessment often involves the identification of the general group of diseases, such as nonsyndromic intellectual disability, epileptic encephalopathy, skeletal dysplasia, inborn error of metabolism, and so on. In some cases, the diagnostic assessment may involve narrow and wide intuition; for instance, there may be a very strong suspicion of a ciliopathy based on clinical findings, with a weaker suspicion of a specific group of ciliopathies, such as Bardet-Biedl syndrome. Current software for supporting diagnostic exome sequencing/genome sequencing does not model the pretest probability (the probability of a specific disease diagnosis before the test is performed). We recently introduced LIRICAL, a likelihood ratio (LR)-based approach for phenotype-driven prioritization,^12^ in which the pretest probability of diseases was modeled uniformly. Another recent algorithm that uses likelihood ratios in phenotype-driven prioritization is Phenotype-driven Likelihood Ratio analysis approach (PheLR)^26^ Here, we extend the LIRICAL algorithm to model clinical intuition, exploiting the ontological representation of diseases of the Mondo ontology.^27^ We show that our approach improves the mean rank of correct candidate diagnoses using simulations. Our approach can be used to model intuition in individual cases or could be applied for cohorts seen in specialty clinics for disease groups such as skeletal dysplasia.

## Materials and Methods

### ClintLR

We introduce an algorithm called Clinical Intuition with Likelihood Ratios (ClintLR) that is available at https://github.com/TheJacksonLaboratory/ClintLR.

### Data

We used Mondo version 2024-07-02 (mondo.json). The 2024-07-01 release of the HPO was used (hp.json and phenotype.hpoa). The HPO resource included 8297 diseases annotated with OMIM identifiers (Supplemental Table 1).

### LIRICAL

The LIRICAL algorithm calculates the LR of observed phenotypic abnormalities and provides estimates of the posttest probability of the candidate diagnoses and the predicted pathogenicity of observed genotypes. LIRICAL version 2.0.0 was used in this study. The algorithm has been described in detail elsewhere.^12^ We review the key mathematical concepts needed to understand the current algorithm in the next section.

### Likelihood ratios and pre/posttest probability

The LR is defined as the probability of an observed value x (eg, an individual test result) in the presence of disease D divided by the probability of that same value in the absence of disease (¬D):

(1) $$LR(x)=\frac{Pr(x|D)}{Pr(x|\neg D)}$$

Pr(x|D) is the sensitivity (true-positive rate) of the test, ie, the expected proportion of individuals with disease D who are correctly identified as having x. LIRICAL defines probability distributions to calculate LR values for observed phenotypic abnormalities (HPO terms) and observed genotypes (as inferred from VCF files). For a more detailed discussion of likelihood ratios and the probability distributions as defined in LIRICAL, see reference^12^.

The posttest probability refers to the probability that an individual has a disease D given the information from a vector of test results X and the pretest probability p of the disease. The posttest probability is given by

(2) $$Pr(D|X)=\frac{pLR(X)}{\left(1\;-\;p\right)\;+\;pLR(X)}$$

The value of the pretest probability p depends on the clinical context.^28^ When randomly screening a population for the disease D, for example, p is typically low and can be defined as the population prevalence of the disease. In cases which D is expected to be diagnosed but with some uncertainty, p has intermediate values. Cases with a strong suspicion of the diagnosis D correspond to high values of p.^29^ In the original version of LIRICAL, the pretest probability was assumed to be uniform for all N diseases, ie, p=1/N for each of the N diseases represented in the HPO database.

### Pretest adjustment factor

ClintLR introduces a pretest adjustment factor, a, to model the strength of the clinical intuition that individuals being tested are more likely to present with a disease from a given group of diseases than random chance. If the user has an intuition that k diseases are more likely to be present than chance, the strength of the intuition should be encoded into representation by the factor a>0. Before adjustment, the pretest probability of each disease is $\frac{1}{N}$, given a database with N diseases. The adjustment sets the probability of each of the k diseases to $\frac{1+a}{N}$ and renormalizes all probabilities so that they sum to 1. The adjusted pretest probability of disease d is represented by $p^{a}(d)$.


(3) $$p^{a}(d)=\frac{1\;+\;a}{k\left(1\;+\;a\right)\;+\;N\;-\;k}\approx \frac{1\;+\;a}{N}$$

If a=0, there is no adjustment and ClintLR reverts to the original LIRICAL implementation of uniform pretest probability, $\frac{1}{N}$.

### Evaluation

We curated HPO terms from 787 published case reports. For each case report, we strove to capture all the phenotypic features that were observed or explicitly excluded with corresponding HPO terms. The variants reported in the case reports were recorded using hg38 coordinates. The phenopackets were obtained from Phenopacket Store.^30^

We downloaded the file dbsnp_146.hg38.vcf from the Genome in a Bottle project website.^31^ This file contains dbSNPs for the GRCh38.p2 reference genome. For this analysis, a VCF file was created that contained hg38 exons that were extended by 50 bp in each direction, and the pathogenic variant(s) for each phenopacket were inserted into the VCF file.

## Results

Most current software for phenotype-driven diagnostic support does not take the prevalence or pretest probability of diseases into account. Effectively, each disease to be evaluated is treated as equally likely before performing the analysis. However, there are common clinical situations in which this is not the case. For instance, a specialty clinic for skeletal dysplasias is much more likely to see patients with genetic skeletal disease than patients with other disease groups such as metabolic disease or retinitis pigmentosa. Additionally, even in general medical genetics clinics, the clinician’s first impression often can be used to narrow down the list of diseases that are considered to be likely in the differential diagnosis, although a precise diagnosis cannot be made. For instance, a clinician may suspect a ciliopathy owing to the demonstration of situs inversus and agenesis of the corpus callosum and then order genomic sequencing to search for the precise diagnosis.

**Figure 1:**
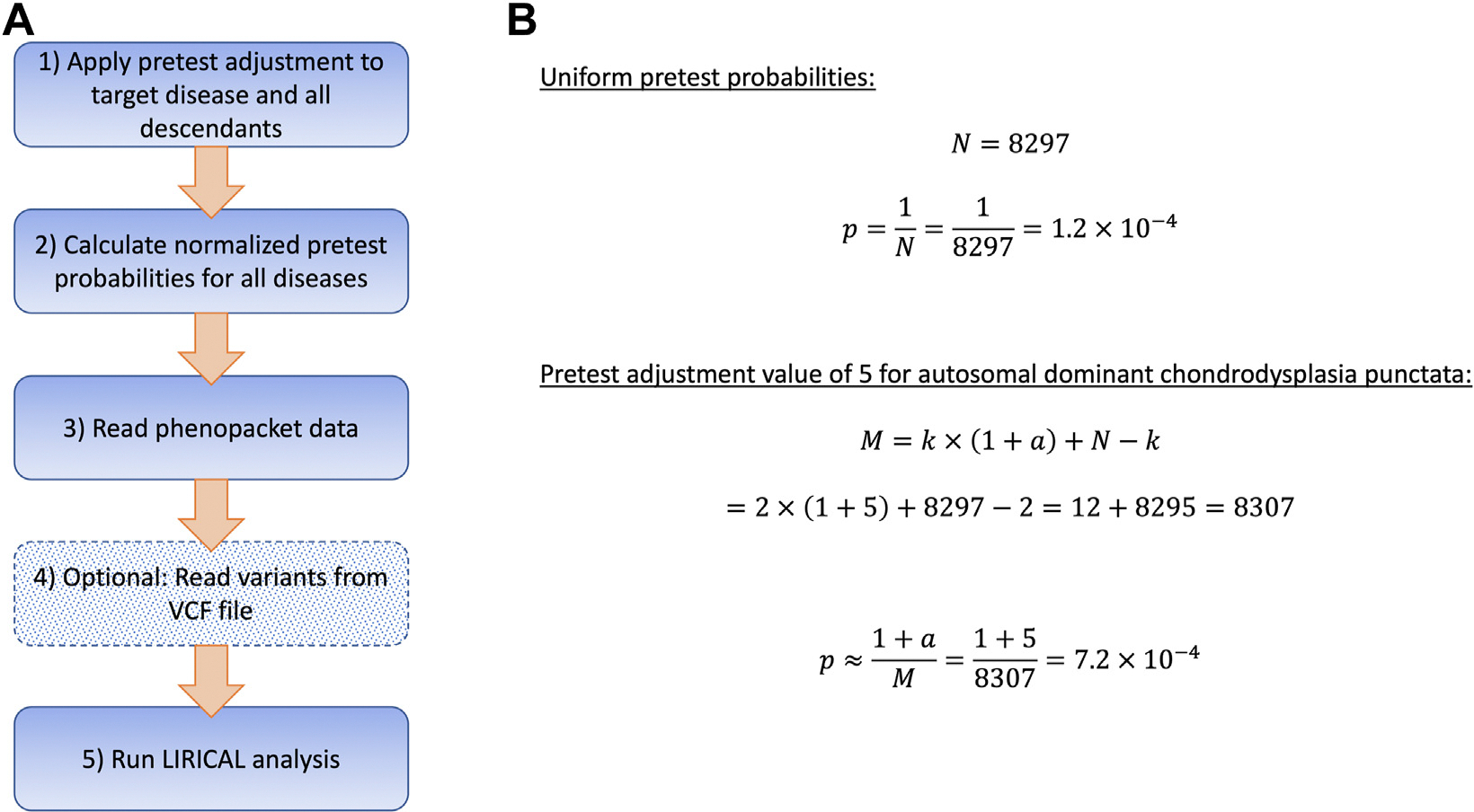
Clinical Inuition with Likelihood Ratios (ClintLR). (A) Flowchart of ClintLR analysis process using adjusted pretest probabilities. (B) Example pretest probability calculations using uniform pretest probability values and a pretest adjustment value of 5 for autosomal dominant chondrodysplasia punctata and its descendants. Applying a pretest value of 5 increases the pretest probability, *p*, from 1.2 × 10^−4^ to 7.2 × 10^−4^.

ClintLR seeks to formalize this situation by adjusting the pretest probability used by the LR-based algorithm LIRICAL. An outline of the analysis process using adjusted pretest probabilities is shown in Figure 1A. Figure 1B shows example calculations for the pretest probabilities using uniform values and a pretest adjustment value of 5 for autosomal dominant chondrodysplasia punctata and its descendants. Using uniform values together with the HPO resource consisting of 8297 disease models, the pretest probability is $\frac{1}{8297}=1.2\;\times \;10^{-4}$ for all diseases.

ClintLR uses a pretest adjustment value to represent the intuition of the user that a disease or group of diseases is more likely to be observed. For instance, given M=8307 diseases and an adjustment of a=5 for the 2 forms of autosomal dominant chondrodysplasia punctata, the pretest probability of each of the specific forms of autosomal dominant chondrodysplasia punctata, eg, chondrodysplasia punctata, tibial-metacarpal type (OMIM:118651; MONDO:0007322), is increased to 7.2 × 10^−4^.

ClintLR requires a pretest probability adjustment value and a phenopacket as inputs and also incorporates various input LIRICAL analysis options, such as the genome build and transcript database, to run the LIRICAL analysis. The paths to the Mondo ontology and LIRICAL data sets are also required to run ClintLR. The ClintLR results are output as an HTML file.

### Benchmarking

To evaluate the performance of ClintLR, we conducted analyses of phenopackets associated with the 787 case reports referenced in the Evaluation section at different levels of clinical intuition. For each case report, the LIRICAL algorithm was executed using uniform pretest probabilities for all diseases, as well as using adjusted pretest probabilities for the target disease included in the phenopacket, along with narrow and broad intuition terms and any descendants. Five different pretest probability adjustment values were used: 1, 5, 10, 15, and 20. For each phenopacket, LIRICAL ranked candidate diseases based on their computed posttest probabilities.

**Figure 2:**
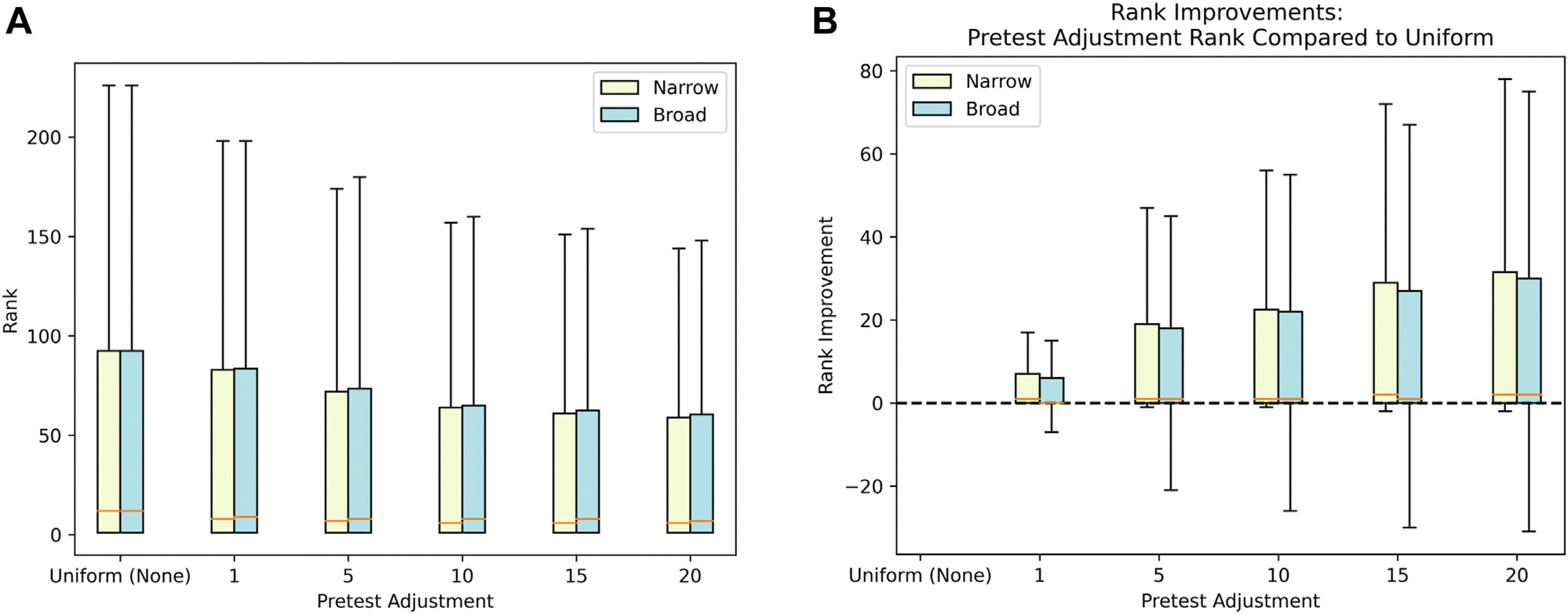
Evaluation. (A) Disease rankings for narrow and broad levels of clinical intuition using 5 pretest adjustment values and uniform pretest probabilities. (B) Disease rank improvements for narrow and broad levels of clinical intuition using 5 pretest adjustment values.

Figure 2A shows the disease rankings for the narrow and broad intuition terms at the 5 pretest adjustment values and using uniform pretest probabilities. Figure 2B shows the improvements in disease rankings for the narrow and broad intuition terms at the 5 pretest adjustment values compared with using uniform pretest probabilities. For both narrow and broad clinical intuition terms, the disease rankings compared with those using uniform pretest probabilities improve as the pretest probability adjustment increases. The improvement in disease rank is slightly higher for narrow intuition terms than it is for broad intuition terms at all 5 pretest adjustment values.

We also tested the disease rankings statistically with the null hypothesis that they are unchanged and the alternative hypothesis that the rankings improve when the pretest adjustment is applied. We performed one-sided Wilcoxon tests for narrow and broad clinical intuition at the 5 pretest adjustment values. Table 1 shows the P values obtained from the Wilcoxon tests. For all pretest adjustment values at both broad and narrow intuition, the P values are less than .05, supporting the alternative hypothesis of an improvement in the disease rankings when the pretest adjustment is applied.

### ClintLR software

**Figure 3:**
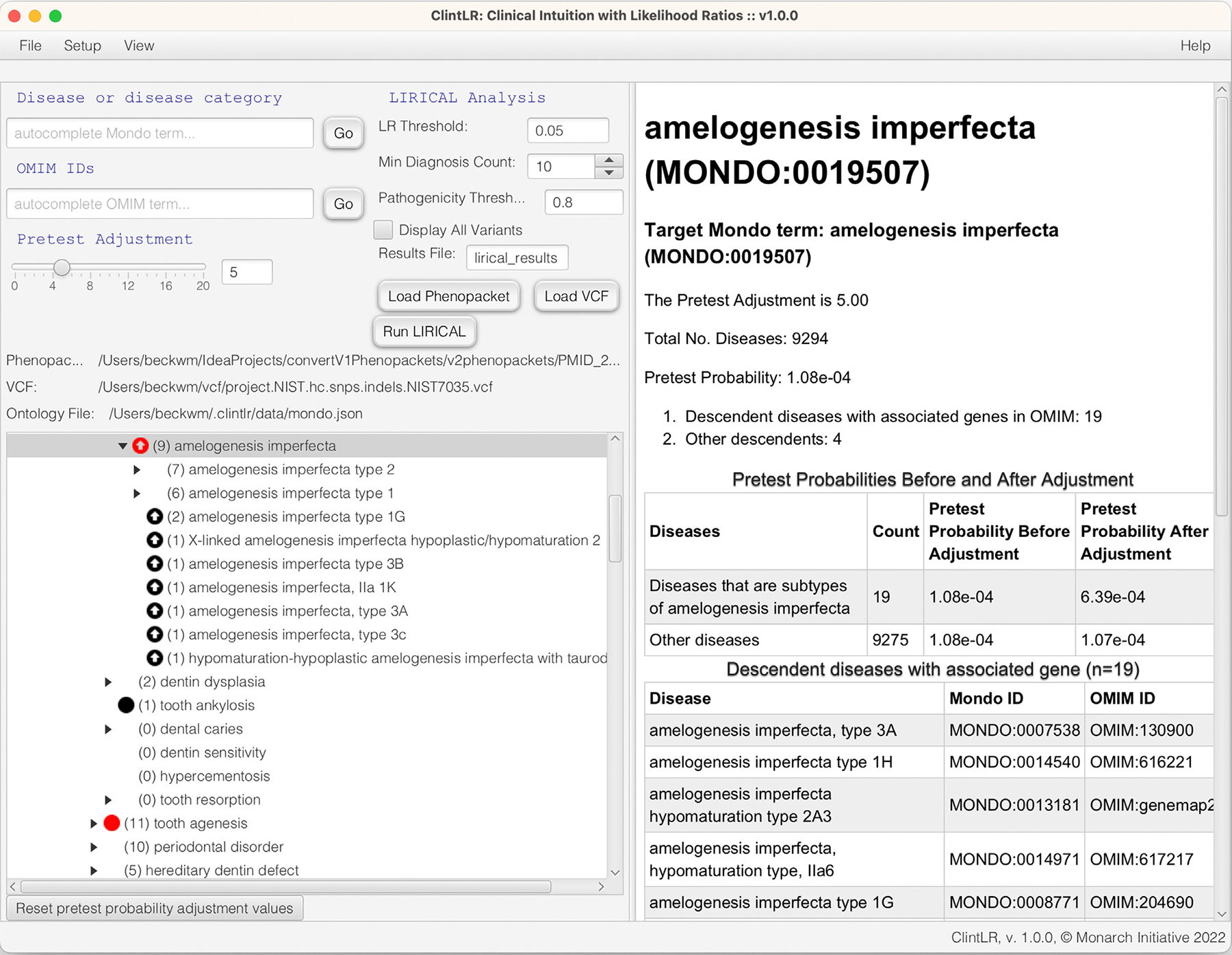
ClintLR Graphical User Interface. The main analysis interface allows users to choose the disease group and customize the pretest adjustment, and thus the pretest probability, of the group to reflect clinical intuition.

We implemented the above described algorithm in a desktop graphical user interface software we call ClintLR. ClintLR displays a tree view of the Mondo Ontology (Figure 3), whereby black circles indicate that a given Mondo term corresponds exactly to an OMIM term, and red circles indicate groups of diseases that correspond to OMIM phenotypic series.^32^ Both Mondo and OMIM terms are fully searchable, and information for the selected term is displayed in an HTML window. There is also a separate window for setting various resources, including paths to the ontology files and LIRICAL settings. The resource setup is persisted between sessions.

Clinical intuition is incorporated into the LIRICAL algorithm by way of a pretest adjustment slider in the ClintLR interface. The slider increases the intuition parameter, a, which increases the pretest probability values that get passed into LIRICAL for the selected term and its descendants. In the tree, an up arrow in the selected term’s icon indicates that the pretest probability has been increased for that term. The pretest probability adjustment values can be viewed in a separate window. If desired, the pretest probability values can be saved as a separate text file, which can then be loaded later.

**Figure 4:**
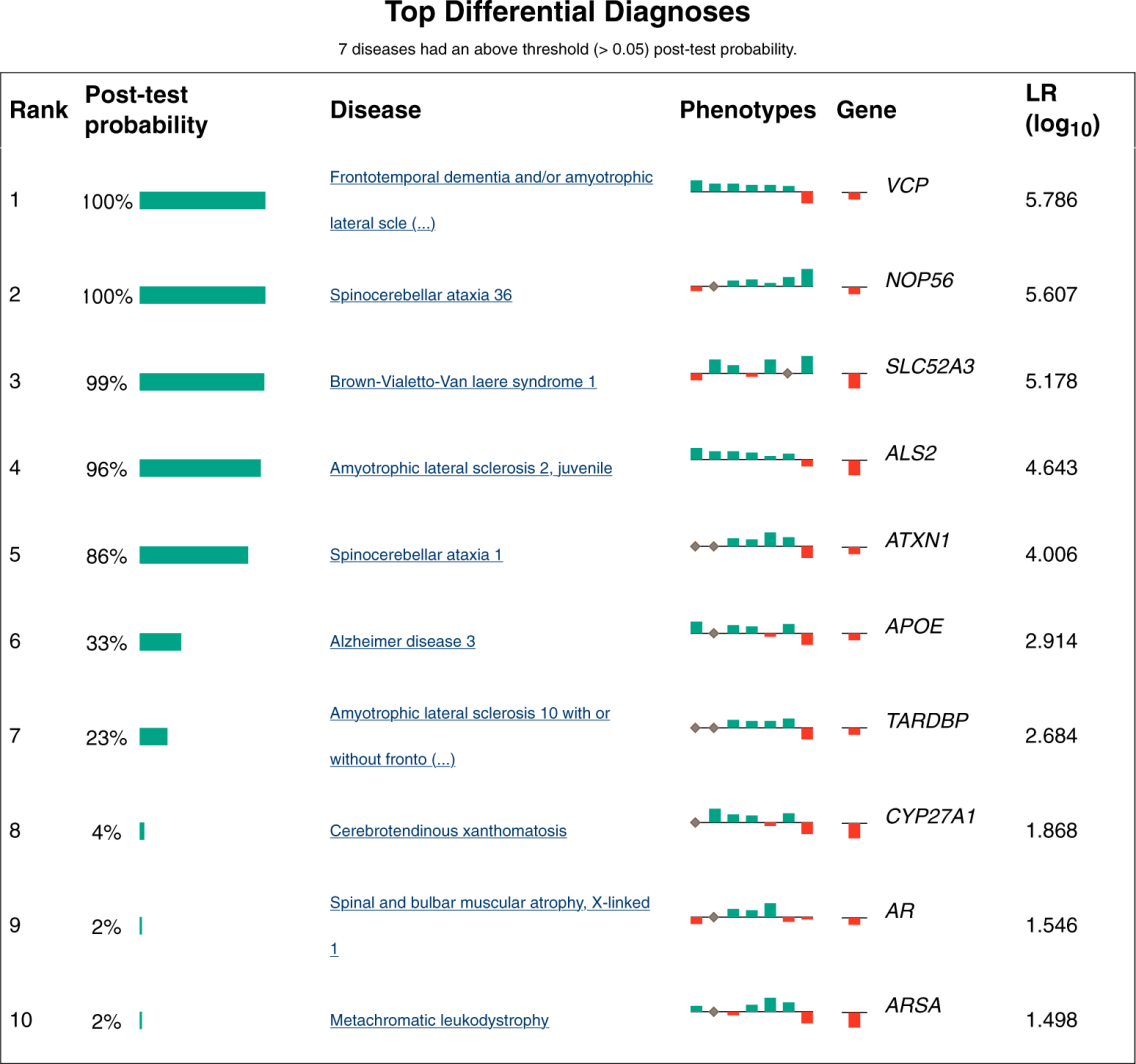
ClintLR runs LIRICAL with the adjusted pretest probabilities and displays the results in the system browser. The posttest probability of the differential diagnosis is reported along with a horizontal green bar that represents its magnitude. The phenotypic features that support the differential diagnosis are shown as green bars above the baseline whose height represents the magnitude of the likelihood ratio. Similarly, features that speak against the differential diagnosis are shown as red bars below the baseline. The genotype likelihood ratio is presented along with the phenotypic features.

Users can set the path to a GA4GH phenopacket with data about an individual for whom the differential diagnosis is being performed, and ClintLR will run LIRICAL using the adjusted pretest probabilities. The LIRICAL analysis can be performed using only phenotypic information or using both phenotypic + genotypic information by loading a VCF file into ClintLR before running LIRICAL. The results of the analysis will be presented in a new HTML file that is opened in the system browser (Figure 4). The html file of the results is also saved separately locally.

## Discussion

Currently, most software for phenotype-driven NGS diagnostic support in rare-disease genomics implicitly assumes a uniform disease pretest probability for each of the tested diseases or disease genes. For instance, if the software prioritizes disease genes, then each of the genes associated with at least 1 Mendelian disease is assumed to be equally likely (currently, 4680 distinct genes are associated with at least one Mendelian disease in the HPO Annotations resource). Similarly, if software prioritizes diseases, then each disease is assumed to be equally likely. Although this assumption is rarely mentioned in the methods, it was explicitly described in the original version of the LIRICAL tool.^12^

However, in some cases the likelihood of a particular disease getting diagnosed is higher than it would be in the general population, such as at specialty clinics. For instance, a skeletal dysplasia clinic is more likely to investigate a patient with a genetic skeletal disease than with a genodermatosis or cystic fibrosis. Even in general genetics clinics, the clinician’s initial clinical impression often can be used to pick out the general group of diseases as a starting point in lieu of a specific disease diagnosis. ClintLR is well suited in both of these scenarios because it incorporates clinical intuition into the LIRICAL algorithm.

We tested ClintLR in 2 scenarios. With “narrow,” we chose a disease group closely related to the original diagnosis. For instance, if the actual diagnosis was Bardet-Biedl syndrome 4, we chose the group ^“^Bardet-Biedl syndrome^”^ (MONDO:0015229), which includes 22 diseases. Here, there was a relatively substantial effect on the average improvement of the rank of the correct disease. With the ^“^broad^”^ scenario, we chose larger groups, such as ^“^ciliopathy^”^ (MONDO:0005308). The ciliopathy group includes the Bardet-Biedl syndromes but also 163 additional diseases. We suggest that improvements seen in the narrow scenario may be reflective of those to be expected when using ClintLR in situations which there is a strong clinical differential diagnosis that indicates that the true diagnosis must be one of a small number of diseases, and the results seen in the broad scenario are likely to be representative of the performance if the differential diagnosis is less clear. Prospective clinical studies would be needed to determine the actual benefit of ClintLR in typical clinical situations.

A limitation of ClintLR is that the LIRICAL software uses OMIM identifiers to specify diseases, whereas the grouping of diseases in the ClintLR software is done with help of the hierarchical structure of Mondo. The mapping of OMIM to Mondo terms did not work in a small number of cases, leading to a failure of ClintLR to prioritize the correct disease. The Mondo ontology is a work in progress, and the hierarchical structure of Mendelian diseases has been under revision. Some of the diseases tested in this work could not be placed under distinct narrow and broad categories. In some cases this was due to the fact that the disease in question does not belong to a recognized disease group, but in others the relevant links in Mondo were missing. We expect that the performance of ClintLR will improve hand in hand with improvements to Mondo.

Another limitation is that if the clinical intuition used to run ClintLR is wrong, then the correct diagnosis may be ranked in a lower position. In cases which a promising candidate diagnosis cannot be found by ClintLR analysis, we advise users to try using a range of settings for the pretest adjustment slider, including 0 (which is equivalent to using the original LIRICAL implementation) or other software for diagnostic support. Finally, ClintLR, as with all software that depends on phenotypic data derived from the HPO, depends on the accuracy and comprehensiveness of the data in the HPO. Suggestions for corrections or additional HPO terms or annotations from the community are always welcome and will serve to improve the performance of HPO-based software for all.

## Conclusion

ClintLR provides a simple method for incorporating clinical intuition in computational differential diagnostic support. An open-source desktop implementation of ClintLR is provided that allows users to choose a group of diseases that reflect their clinical intuition as to the differential diagnosis, set an adjustment factor to reflect the strength of their clinical impression, and to run the LIRICAL algorithm with adjusted pretest probabilities. Our benchmarking shows a substantial and significant improvement of ranking of the correct diagnosis.

## Supplementary Material

Supplemental Table S1

## Figures and Tables

**Table 1:** *P* values for Wilcoxon Tests for narrow and broad clinical intuition at 5 pretest adjustment values

	Wilcoxon Test *P* Value	
Pretest Adjustment	Narrow Intuition	Broad Intuition

1	9.8 × 10^−70^	4.4 × 10^−64^
5	1.6 × 10^−75^	2.5 × 10^−70^
10	4.5 × 10^−76^	2.8 × 10^−71^
15	5.8 × 10^−76^	9.0 × 10^−71^
20	1.1 × 10^−76^	2.1 × 10^−71^

## Data Availability

The ClintLR source code, executable, and documentation are freely available under an MIT license at https://github.com/TheJacksonLaboratory/ClintLR. Release 1.0.0 corresponds to the version used in this manuscript. The 787 GA4GH phenopackets used for the evaluation are available at https://zenodo.org/records/13285992.

## References

[1] Marwaha S, Knowles JW, Ashley EA. A guide for the diagnosis of rare and undiagnosed disease: beyond the exome. Genome Med. 2022;14(1):23. 10.1186/s13073-022-01026-w 35220969 PMC8883622

[2] de Haan A, Eijgelsheim M, Vogt L, Knoers NVAM, de Borst MH. Diagnostic yield of next-generation sequencing in patients with chronic kidney disease of unknown etiology. Front Genet. 2019;10:1264. 10.3389/fgene.2019.01264 31921302 PMC6923268

[3] Bertoli-Avella AM, Beetz C, Ameziane N, Successful application of genome sequencing in a diagnostic setting: 1007 index cases from a clinically heterogeneous cohort. Eur J Hum Genet. 2021;29(1):141–153. 10.1038/s41431-020-00713-9 32860008 PMC7852664

[4] Souche E, Beltran S, Brosens E, Recommendations for whole genome sequencing in diagnostics for rare diseases. Eur J Hum Genet. 2022;30(9):1017–1021. 10.1038/s41431-022-01113-x 35577938 PMC9437083

[5] Yépez VA, Gusic M, Kopajtich R, Clinical implementation of RNA sequencing for Mendelian disease diagnostics. Genome Med. 2022;14(1):38. 10.1186/s13073-022-01019-9 35379322 PMC8981716

[6] Sahajpal NS, Mondal AK, Fee T, Clinical validation and diagnostic utility of optical genome mapping in prenatal diagnostic testing. J Mol Diagn. 2023;25(4):234–246. 10.1016/j.jmoldx.2023.01.006 36758723

[7] Ahsan MU, Liu Q, Perdomo JE, Fang L, Wang K. A survey of algorithms for the detection of genomic structural variants from long-read sequencing data. Nat Methods. 2023;20(8):1143–1158. 10.1038/s41592-023-01932-w 37386186 PMC11208083

[8] Smedley D, Robinson PN. Phenotype-driven strategies for exome prioritization of human Mendelian disease genes. Genome Med. 2015;7(1):81. 10.1186/s13073-015-0199-2 26229552 PMC4520011

[9] Jacobsen JOB, Kelly C, Cipriani V, Phenotype-driven approaches to enhance variant prioritization and diagnosis of rare disease. Hum Mutat. 2022;43(8):1071–1081. 10.1002/humu.24380 35391505 PMC9288531

[10] Kelly C, Szabo A, Pontikos N, Phenotype-aware prioritisation of rare Mendelian disease variants. Trends Genet. 2022;38(12):1271–1283. 10.1016/j.tig.2022.07.002 35934592 PMC9950798

[11] Robinson PN, Köhler S, Oellrich A, Improved exome prioritization of disease genes through cross-species phenotype comparison. Genome Res. 2014;24(2):340–348. 10.1101/gr.160325.113 24162188 PMC3912424

[12] Robinson PN, Ravanmehr V, Jacobsen JOB, Interpretable clinical genomics with a likelihood ratio paradigm. Am J Hum Genet. 2020;107(3):403–417. 10.1016/j.ajhg.2020.06.021 32755546 PMC7477017

[13] Zemojtel T, Köhler S, Mackenroth L, Effective diagnosis of genetic disease by computational phenotype analysis of the disease-associated genome. Sci Transl Med. 2014;6(252):252ra123. 10.1126/scitranslmed.3009262 PMC4512639 25186178

[14] Li Q, Zhao K, Bustamante CD, Ma X, Wong WH. Xrare: a machine learning method jointly modeling phenotypes and genetic evidence for rare disease diagnosis. Genet Med. 2019;21(9):2126–2134. 10.1038/s41436-019-0439-8 30675030 PMC6752318

[15] Anderson D, Baynam G, Blackwell JM, Lassmann T. Personalised analytics for rare disease diagnostics. Nat Commun. 2019;10(1):5274. 10.1038/s41467-019-13345-5 31754101 PMC6872807

[16] Wu C, Devkota B, Evans P, Rapid and accurate interpretation of clinical exomes using Phenoxome: a computational phenotype-driven approach. Eur J Hum Genet. 2019;27(4):612–620. 10.1038/s41431-018-0328-7 30626929 PMC6460638

[17] Boudellioua I, Kulmanov M, Schofield PN, Gkoutos GV, Hoehndorf R. DeepPVP: phenotype-based prioritization of causative variants using deep learning. BMC Bioinformatics. 2019;20(1):65. 10.1186/s12859-019-2633-8 30727941 PMC6364462

[18] Gall T, Valkanas E, Bello C, Defining disease, diagnosis, and translational medicine within a homeostatic perturbation paradigm: the National Institutes of Health undiagnosed diseases program experience. Front Med (Lausanne). 2017;4:62. 10.3389/fmed.2017.00062 28603714 PMC5445140

[19] Beaulieu CL, Majewski J, Schwartzentruber J, FORGE Canada Consortium: outcomes of a 2-year national rare-disease gene-discovery project. Am J Hum Genet. 2014;94(6):809–817. 10.1016/j.ajhg.2014.05.003 24906018 PMC4121481

[20] Thompson R, Johnston L, Taruscio D, RD-Connect: an integrated platform connecting databases, registries, biobanks and clinical bioinformatics for rare disease research. J Gen Intern Med. 2014;29(suppl 3):S780–S787. 10.1007/s11606-014-2908-8 25029978 PMC4124112

[21] Boycott KM, Rath A, Chong JX, International cooperation to enable the diagnosis of all rare genetic diseases. Am J Hum Genet. 2017;100(5):695–705. 10.1016/j.ajhg.2017.04.003 28475856 PMC5420351

[22] Köhler S, Doelken SC, Mungall CJ, The Human Phenotype Ontology project: linking molecular biology and disease through phenotype data. Nucleic Acids Res. 2014;42(database issue):D966–D974. 10.1093/nar/gkt1026 24217912 PMC3965098

[23] Köhler S, Vasilevsky NA, Engelstad M, The human phenotype ontology in 2017. Nucleic Acids Res. 2017;45(D1):D865–D876. 10.1093/nar/gkw1039 27899602 PMC5210535

[24] Köhler S, Carmody L, Vasilevsky N, Expansion of the Human Phenotype Ontology (HPO) knowledge base and resources. Nucleic Acids Res. 2019;47(D1):D1018–D1027. 10.1093/nar/gky1105 30476213 PMC6324074

[25] Gargano MA, Matentzoglu N, Coleman B, The Human Phenotype Ontology in 2024: phenotypes around the world. Nucleic Acids Res. 2024;52(D1):D1333–D1346. 10.1093/nar/gkad1005 37953324 PMC10767975

[26] Yang J, Shu L, Duan H, Li H. A robust phenotype-driven likelihood ratio analysis approach assisting interpretable clinical diagnosis of rare diseases. J Biomed Inform. 2023;142(104372). 10.1016/j.jbi.2023.104372 37105510

[27] Putman TE, Schaper K, Matentzoglu N, The monarch Initiative in 2024: an analytic platform integrating phenotypes, genes and diseases across species. Nucleic Acids Res. 2024;52(D1):D938–D949. 10.1093/nar/gkad1082 38000386 PMC10767791

[28] van der Helm HJ, Hische EA. Application of Bayes’s theorem to results of quantitative clinical chemical determinations. Clin Chem. 1979;25(6):985–988. 10.1093/clinchem/25.6.985 445835

[29] Albert A On the use and computation of likelihood ratios in clinical chemistry. Clin Chem. 1982;28(5):1113–1119. 10.1093/clinchem/28.5.1113 7074890

[30] Danis D, Bamshad MJ, Bridges Y, A corpus of GA4GH phenopackets: case-level phenotyping for genomic diagnostics and discovery. HGG Adv. 2024:100371. 10.1016/j.xhgg.2024.100371 39394689 PMC11564936

[31] Zook JM, Catoe D, McDaniel J, Extensive sequencing of seven human genomes to characterize benchmark reference materials. Sci Data. 2016;3(1):160025. 10.1038/sdata.2016.25 27271295 PMC4896128

[32] Amberger JS, Bocchini CA, Scott AF, Hamosh A. OMIM.org: leveraging knowledge across phenotype-gene relationships. Nucleic Acids Res. 2019;47(D1):D1038–D1043. 10.1093/nar/gky1151 30445645 PMC6323937

